# Hematological Disease Profile in the Tribal Population of Northern Maharashtra Based on Bone Marrow Examination: A Retrospective Study

**DOI:** 10.7759/cureus.100956

**Published:** 2026-01-06

**Authors:** Ashwini Khadatkar, Shivam Rainchwar, Amit P Gujarathi

**Affiliations:** 1 Department of Pathology, SMBT Institute of Medical Sciences and Research Centre, Nashik, IND; 2 Department of Community Medicine, SMBT Institute of Medical Sciences and Research Centre, Nashik, IND

**Keywords:** anemia, bone marrow examination, hematologic disorders, myelodysplastic syndrome, myeloproliferative neoplasms, peripheral smear, plasma cell dyscrasias, tribal population

## Abstract

Background

Bone marrow examination (BME), comprising bone marrow aspiration (BMA) and bone marrow biopsy (BMB), remains vital for diagnosing hematological disorders, especially when peripheral blood findings are inconclusive. Data on marrow pathology among India’s tribal population are limited. Hence, this study aimed to assess clinical indications, diagnostic utility, and association between peripheral blood smear (PBS) and bone marrow findings in the tribal population of northern Maharashtra.

Methodology

This retrospective, descriptive study included 60 tribal patients who underwent BMA and BMB between January 2023 and March 2024. After excluding inadequate and normal samples, 39 cases were analyzed. Demographic, clinical, and morphological data were reviewed. Association between PBS and bone marrow findings was tested using the Z-test for proportions (p < 0.05).

Results

Among 60 tribal patients who underwent BME, 39 (65%) yielded adequate and interpretable findings for analysis. Anemia was the most common indication, observed in 23 (59.0%) patients, including microcytic 11 (28.2%), megaloblastic 9 (23.1%), and dimorphic 3 (7.7%) cases. Bone marrow showed a significantly higher diagnostic yield in microcytic anemia compared with PBS (79.0% vs. 21.0%; p < 0.01). Myelodysplastic syndrome (5, 12.8%) was detected exclusively on marrow (p = 0.002). Myeloproliferative neoplasms (5, 12.8%), plasma cell dyscrasias (4, 10.3%), and immune thrombocytopenic purpura (2, 5.1%) were also identified.

Conclusions

BME demonstrated superior diagnostic accuracy over peripheral smear, particularly for anemia and myelodysplastic syndromes. Strengthening hematopathology services in tribal regions can facilitate earlier diagnosis and management of reversible hematologic disorders, improving outcomes in underserved populations.

## Introduction

Bone marrow examination (BME), including bone marrow aspiration (BMA) and trephine bone marrow biopsy (BMB), is an indispensable diagnostic tool in the evaluation of a broad spectrum of hematological and non-hematological disorders [1-3]. It is particularly valuable when peripheral blood studies are inconclusive and remains essential for staging and monitoring various malignancies [1,3,4]. While BMA provides cytological detail and rapid diagnosis of conditions such as acute leukemia, BMB offers architectural and histological insights into marrow cellularity, fibrosis, and infiltration, especially in cases of dry taps, aplastic anemia, and infiltrative lesions [3-7].

The common indications for BME include unexplained cytopenias, pancytopenia, anemia, suspected hematological malignancies, pyrexia of unknown origin, thrombocytopenia, and organomegaly [1-6,8-10]. Among non-malignant disorders, anemia, particularly megaloblastic and microcytic types, constitutes a major diagnostic category, reflecting underlying nutritional deficiencies and infections [2,5,7,9]. Malignant hematological conditions such as acute leukemia, myelodysplastic syndromes (MDSs), myeloproliferative neoplasms (MPNs), and plasma cell dyscrasia (PCDs) are also frequently identified through marrow studies [2-6,9,11-13].

Evidence from epidemiological studies highlights a substantial burden of non-neoplastic disorders diagnosed through BME. In a large cross-sectional study from Northeast Ethiopia, non-neoplastic conditions constituted 57.0% of bone marrow diagnoses, with erythroid hyperplasia (24.6%), aplastic anemia (10.5%), megaloblastic anemia (7.0%), and iron deficiency-related anemia (1.8%) being the most common conditions [14]. Neoplastic disorders accounted for 11.4% of cases, predominantly chronic myeloid leukemia (3.5%), acute myeloid leukemia (2.6%), chronic lymphocytic leukemia (1.8%), multiple myeloma (1.8%), and acute lymphoblastic leukemia (0.9%) [14]. Similar South Asian studies have reported non-neoplastic disorders as the dominant diagnostic spectrum, while hematological malignancies comprise approximately 10-15% of bone marrow diagnoses [15,16].

Despite its diagnostic value, discrepancies can occur between BMA and BMB findings, underscoring the importance of performing both procedures to improve diagnostic accuracy [3,6,7,10]. Such discordance is common in cases of fibrosis, hypoplasia, or patchy marrow infiltration.

Most studies evaluating BMEs have been conducted in urban tertiary centers, with limited data focusing on tribal populations, who often face nutritional deprivation, limited healthcare access, and delayed diagnosis. Despite the documented burden of both non-neoplastic and neoplastic hematological disorders in other regions, the spectrum and pattern of marrow pathology in tribal communities of India remain underexplored.

Hence, this study was undertaken to evaluate the indications for BMA and BMB, determine their diagnostic utility, and analyze the correlation between peripheral smear and bone marrow findings in the tribal population of northern Maharashtra.

## Materials and methods

Study design and setting

This retrospective, descriptive study was conducted in the Department of Pathology at a tertiary care teaching hospital located in a predominantly tribal, resource-limited region of northern Maharashtra, India. The study aimed to evaluate the indications for BME, determine its diagnostic utility, and assess concordance between peripheral blood smear (PBS) findings and bone marrow morphology in the tribal population. Data were retrieved from the Department of Pathology registries and laboratory records over a 15-month period from January 1, 2023, to March 31, 2024. All tribal patients who underwent both BMA and BMB during this period were reviewed.

Study population and eligibility criteria

Cases were included if registry-based pathological data, including PBS reports and interpretable BMA and BMB findings, were available. Although clinical records were accessible for all included cases, detailed biochemical parameters such as iron studies, vitamin B12, and folate levels were not consistently available due to the retrospective design and the resource-limited setting.

All consecutive tribal patients who underwent BMA and trephine BMB during the study period were initially included. Cases were excluded from disease-specific diagnostic categorization if bone marrow aspirates were inadequate or diluted, biopsy cores were poorly preserved or non-interpretable, or if marrow examination revealed normocellular morphology without evidence of a specific hematological pathology.

Of the 60 BMEs reviewed during the study period, 10 cases were excluded due to inadequate or technically suboptimal samples, while 11 cases demonstrated normocellular marrow without a definable pathological diagnosis. Although these cases were included in the overall study cohort, they were excluded from disease-specific analysis as definitive diagnostic categorization was not possible. After excluding these 21 cases, 39 (65%) cases constituted the final evaluable cohort for diagnostic and statistical analysis. All subsequent frequencies and proportions were calculated based on this evaluable subset unless otherwise specified.

Peripheral blood smear and bone marrow examination

Peripheral blood samples were collected in ethylenediaminetetraacetic acid vacutainers, and smears were prepared and stained using Leishman stain. Morphological evaluation included red blood cell morphology, differential leukocyte count, platelet assessment, and identification of abnormal cells or blasts. Slides were examined under oil immersion (1,000×) using a Leica DM3000 microscope.

BMA and BMB were performed under aseptic precautions, usually from the posterior superior iliac spine, using a Salah aspiration needle (2.0 mm × 50 mm) and a Jamshidi biopsy needle (11 G × 100 mm). Aspirates (0.5-1.0 mL) were stained with May-Grunwald-Giemsa and Leishman stains. Biopsy specimens were fixed in 10% neutral buffered formalin, decalcified using 10% formic acid, embedded in paraffin, and sectioned at 3-5 µm. Sections were stained with hematoxylin and eosin. Special stains, including reticulin (Gomori silver) and periodic acid-Schiff, were performed when indicated. Due to the retrospective nature of the study, representative images of stained slides were not consistently available.

Diagnostic criteria and categorization

All cases were diagnosed and categorized based on bone marrow morphology in accordance with the World Health Organization (WHO) 2016 classification. [17,18] Anemias were classified morphologically into microcytic, dimorphic, and megaloblastic anemia.

MDSs were diagnosed based on the presence of dysplasia in ≥10% of cells in one or more hematopoietic lineages. PCDs were diagnosed when plasma cells constituted ≥10% of bone marrow cellularity. MPNs were assessed based on overall marrow cellularity and lineage expansion.

BMB, when adequate, was considered the reference (gold) standard for final diagnosis, in accordance with WHO recommendations for the evaluation of hematological disorders. PBS and BMA findings were interpreted in relation to trephine biopsy morphology to assess diagnostic association. A sequential evaluation approach (PBS → BMA → BMB) was employed to reflect routine diagnostic practice in resource-limited settings. This approach was used for pragmatic assessment and was not intended to imply diagnostic superiority. In cases with discordant findings, the final diagnosis was based on trephine biopsy morphology after review by senior pathologists.

Statistical analysis

Categorical variables were summarized as frequencies and percentages. Association between PBS findings and bone marrow diagnoses was assessed using the Z-test for difference in proportions, with a p-value <0.05 considered statistically significant. Statistical analysis was performed using SPSS Statistics for Windows, version 25.0 (IBM Corp., Armonk, NY, USA).

Ethical approval

The study was approved by the Institutional Ethics Committee (approval number: 1401/SMBT/IMSRC/10/IEC/23/169). As the study utilized retrospective, anonymized registry-based data, the requirement for informed consent was waived. Confidentiality and ethical standards were maintained throughout the study.

## Results

Table 1 presents the age- and sex-wise distribution of the 60 patients who underwent BME during the study period. The study population comprised 35 (58.3%) males and 25 (41.7%) females. The most commonly affected age group was 41-50 years, accounting for 50.0% (n = 30) of the total cases, followed by the 21-30-year age group at 30.0% (n = 18). The youngest age group (10-20 years) constituted 13.3% (n = 8) of cases, while patients aged 61-70 years represented 6.7% (n = 4) of the study population. The findings indicate a predominance of BMEs in middle-aged adults, with a slight male preponderance. This age distribution reflects the higher burden of hematological disorders requiring marrow evaluation in the economically productive age group within this tribal population.

**Table 1:** Demographic distribution (age and gender) of patients who underwent bone marrow examination.

Age group (years)	Male, n (%)	Female, n (%)	Total, n (%)
10–20	4 (11.4)	4 (16.0)	8 (13.3)
21–30	13 (37.1)	5 (20.0)	18 (30.0)
41–50	16 (45.7)	14 (56.0)	30 (50.0)
61–70	2 (5.7)	2 (8.0)	4 (6.7)
Total	35 (100.0)	25 (100.0)	60 (100.0)

As shown in Table 2, BME demonstrated a higher diagnostic yield than PBS for all anemia subtypes. This difference was statistically significant for microcytic anemia (Z = 3.02, p < 0.01), whereas differences observed in megaloblastic and dimorphic anemia were not statistically significant. Cases with near-normal marrow and mild erythroid hyperplasia could not be reliably categorized based on peripheral smear findings and were excluded from disease-specific analysis.

**Table 2:** Comparison of bone marrow examination with peripheral smear findings in anemia (n = 23). Cases with near-normal marrow and mild erythroid hyperplasia were part of the overall cohort (N = 60) but were excluded from disease-specific diagnostic and statistical analysis due to the absence of a definitive pathological diagnosis. Percentages for anemia subtypes are calculated from the evaluable cohort (n = 39). The Z-test was used to compare proportions; *: p-values <0.05 are considered statistically significant. PBS: peripheral blood smear; BMA: bone marrow aspiration; BMB: bone marrow biopsy; EHP: erythroid hyperplasia

Diagnosis	Peripheral smear (PBS)	BMA	BMB	Detected by BMA/BMB, n (%)	Detected by PBS, n (%)	Z-value	P-value
Microcytic anemia	Microcytes + normocytes	Hypercellular with EHP; erythroid hyperplasia (micronormoblastic)	Hypercellular marrow with EHP and micronormoblastic pattern	11 (79.0%)	3 (21.0%)	3.02	<0.01*
Megaloblastic anemia	Macrocytes, ovalocytes, and hypersegmented neutrophils	Hypercellular with EHP; erythroid hyperplasia (macronormoblastic)	Hypercellular marrow with EHP and macronormoblastic erythroid series	9 (64.3%)	5 (35.7%)	1.51	>0.05
Dimorphic anemia	Mixed microcytic + macrocytic + normocytic cells	Hypercellular with EHP; mixed micronormo and macro pattern	Hypercellular marrow with dual erythroid proliferation	3 (60.0%)	2 (40.0%)	0.63	>0.05
Near-normal marrow with mild EHP	Normocytes with mild hypochromia	Mild EHP	Mild EHP	11 (18.3%)	–	–	–

Morphologically, microcytic anemia revealed microcytes and normocytes on PBS, while the marrow was hypercellular with micronormoblastic erythroid hyperplasia. In megaloblastic anemia, PBS showed macrocytes, ovalocytes, and hypersegmented neutrophils; marrow confirmed macronormoblastic erythroid hyperplasia. Dimorphic anemia displayed a dual red cell population on PBS, corresponding to mixed normoblastic and megaloblastic proliferation in the marrow.

Table 3 compares PBS findings with BMA and BMB findings in cases of MPNs. In essential thrombocythemia, equal diagnostic detection was observed on PBS and BME (50% each). In polycythemia vera, the diagnosis was established exclusively on BME, with no cases identified on peripheral smear; however, this difference was not statistically significant (p = 0.157). For chronic myeloid leukemia, equal detection rates were observed on PBS and BME (50% each).

**Table 3:** Comparison of bone marrow examination and peripheral smear findings in myeloproliferative neoplasms (n = 5). The Z-test was used to compare proportions. Statistical results should be interpreted with caution due to small subgroup sizes PBS: peripheral blood smear; BMA: bone marrow aspiration; BMB: bone marrow biopsy; EHP: erythroid hyperplasia

Diagnosis	PBS	BMA	BMB	BMA/BMB, n (%)	PBS, n (%)	Z-value	P-value
Essential thrombocythemia	Normocytic with mild hypochromia	Hypercellular with EHP and megakaryocytic proliferation	Hypercellular erythroid hyperplasia with increased megakaryocytes	1 (50.0%)	1 (50.0%)	0.00	1.000
Polycythemia vera	Normocytic with mild hypochromia	Hypercellular with EHP and erythroid hyperplasia	Hypercellular erythroid marrow	1 (100.0%)	0 (0.0%)	1.41	0.157
Chronic myeloid leukemia	Normocytic with mild hypochromia	Hypercellular with EHP and few blasts	Hypercellular marrow with myeloid predominance and rare blasts	3 (50.0%)	3 (50.0%)	0.00	1.000

Morphologically, PBS in MPN cases commonly showed normocytic cells with mild hypochromia, whereas bone marrow demonstrated hypercellular marrow with erythroid hyperplasia. Chronic myeloid leukemia cases showed myeloid proliferation with few blasts, while essential thrombocythemia and polycythemia vera exhibited erythroid hyperplasia and megakaryocytic prominence.

Table 4 compares the diagnostic yield of PBS with BMA and BMB in selected hematological disorders. In cases of immune thrombocytopenic purpura, BME detected a higher proportion of cases (66.7%) compared to peripheral smear (33.3%), though this difference was not statistically significant (p = 0.414).

**Table 4:** Comparison of bone marrow examination and peripheral smear findings in other blood dyscrasias (n = 11). The Z-test was used to compare proportions; *: p-values <0.05 are considered statistically significant. Statistical results should be interpreted cautiously due to small subgroup sizes. PBS: peripheral blood smear; BMA: bone marrow aspiration; BMB: bone marrow biopsy; EHP: erythroid hyperplasia

Diagnosis	PBS	BMA	BMB	BMA/BMB, n (%)	PBS, n (%)	Z-value	P-value
Immune thrombocytopenic purpura	Platelets ↓; no megakaryocytes	Near normocellular with ↑ megakaryocytes; mild erythroid hyperplasia	Mild EHP with megakaryocytic increase	2 (66.7%)	1 (33.3%)	0.82	0.414
Myelodysplastic syndrome	Blasts + nucleated red blood cells	Hypercellular with blast cells	Hypercellular marrow with increased blasts and dysplastic changes	5 (100.0%)	0 (0.0%)	3.16	0.002*
Plasma cell dyscrasia/Multiple myeloma	Normocytic cells + plasma cells + rouleaux formation	Hypercellular marrow with plasma cell clusters	Hypercellular plasmacytosis with marrow replacement	4 (57.1%)	3 (42.9%)	0.53	0.593

All cases of MDS were diagnosed on BME (100% detection), whereas none were conclusively identified on peripheral smear alone, resulting in a statistically significant difference (Z = 3.16, p = 0.002). In PCD/multiple myeloma, BME identified a slightly higher proportion of cases (57.1%) compared to peripheral smear (42.9%), without statistical significance. In multiple myeloma, PBS demonstrated plasma cells and rouleaux formation, confirmed by plasma cell proliferation on marrow. Immune thrombocytopenic purpura cases lacked megakaryocytes on PBS but showed normocellular marrow with increased megakaryocytes and mild erythroid hyperplasia.

## Discussion

BME remains a cornerstone for the diagnosis of hematological disorders, particularly in settings where PBS findings are inconclusive or insufficient. In the present retrospective analysis of 60 tribal patients from northern Maharashtra, BMA/BMB provided crucial diagnostic information across a broad spectrum of hematological conditions. The study population was predominantly male (35, 58%), with the highest frequency of cases observed in the 41-50-year age group. This middle-age predominance aligns with earlier Indian studies, which attribute this trend to cumulative nutritional deficiencies, occupational exposures, and delayed healthcare-seeking behavior in rural and tribal regions [6]. The predominance of economically productive age groups also underscores the broader socioeconomic impact of undiagnosed hematological disorders in these communities.

Anemia as the predominant indication

In the present study, anemia constituted the most common indication for BME, accounting for more than two-thirds of cases. Among these, microcytic, megaloblastic, and dimorphic anemias were the dominant subtypes. The predominance of microcytic anemia (11, 28.2%) reflects the persistent burden of iron deficiency in tribal populations, attributable to limited dietary diversity, parasitic infestations, and chronic malnutrition [3]. Megaloblastic and dimorphic anemias, together comprising approximately 30.8% of cases, suggest coexisting folate and vitamin B12 deficiencies, findings that are consistent with other Indian studies conducted in nutritionally vulnerable populations.

A statistically significant difference between BMA/BMB and PBS was observed for microcytic anemia (p < 0.01), confirming the superior diagnostic yield of BME in cases with subtle or mixed morphological patterns on peripheral smear. The detection of erythroid hyperplasia in 38.3% of cases further underscores the marrow’s compensatory response to chronic nutritional deficiency. Similar marrow patterns have been reported by Birare et al., where erythroid hyperplasia with micronormoblastic maturation was the most frequent finding in rural cohorts [13]. These findings reinforce the limitation of PBS alone in accurately characterizing anemia subtypes in resource-limited settings.

Myeloproliferative and myelodysplastic disorders

MPNs and myelodysplastic syndrome (MDS) together accounted for 25.6% of the studied cases. Diagnostic concordance between BMA/BMB and PBS was complete in chronic myeloid leukemia and essential thrombocythemia, whereas polycythemia vera was diagnosed exclusively on BME. This observation reiterates the importance of histological evaluation in conditions where peripheral blood findings may be nonspecific or misleading.

In the case of MDS, all patients (100%) were diagnosed exclusively on BME, with a statistically significant advantage over PBS (p = 0.002). Marrow findings of hypercellularity, increased blasts, and dysplastic changes were consistent with the morphological spectrum described by Howard et al. [19]. This highlights the inability of peripheral smear alone to reliably detect early or low-grade MDS, emphasizing the indispensable role of marrow morphology for accurate diagnosis, risk stratification, and clinical decision-making.

Plasma cell dyscrasias and thrombocytopenic disorders

PCDs (4, 10.3%) and immune thrombocytopenic purpura (2, 5.1%) were less frequent but clinically significant diagnostic categories. In multiple myeloma, PBS revealed plasma cells and rouleaux formation, while BME confirmed plasmacytosis and marrow replacement, findings consistent with the diagnostic criteria outlined by the International Myeloma Working Group [20]. These observations reaffirm that while PBS may raise suspicion, BME remains essential for confirmation and disease burden assessment.

In immune thrombocytopenic purpura, PBS findings were often nondiagnostic due to the absence of megakaryocytes. In contrast, BME demonstrated megakaryocytic hyperplasia, facilitating definitive diagnosis. This underscores the complementary role of marrow evaluation in differentiating peripheral destruction from marrow production defects in thrombocytopenic disorders.

Diagnostic value and clinical utility

Overall, only 30% of cases demonstrated complete clinical-hematological association, a finding comparable to reports from other resource-limited settings in India [9]. This highlights the inherent limitations of relying solely on clinical features and peripheral smear findings for definitive diagnosis. Thus, BME serves a dual role: confirming preliminary diagnoses and uncovering treatable or unsuspected conditions, including nutritional anemias, early MDS, and PCDs, which may otherwise remain undiagnosed.

Relevance to the tribal population

The study provides valuable insights into hematological disease patterns in a marginalized tribal population, where undernutrition, chronic infections, and restricted access to healthcare contribute to delayed diagnosis and treatment. Early and systematic utilization of BME in selected cases can significantly enhance diagnostic accuracy and guide appropriate therapy, particularly for reversible conditions. Strengthening hematology diagnostic services at secondary-level healthcare facilities could potentially reduce morbidity associated with nutritional and clonal marrow disorders in these communities.

Limitations and future scope

As a retrospective, single-center study, this analysis was limited by the unavailability of ancillary investigations such as serum ferritin, vitamin B12, folate assays, cytogenetic, and molecular studies. Additionally, the absence of uniformly available slide images is an inherent limitation of retrospective pathology-based studies. Future multicentric prospective studies integrating morphological, biochemical, and molecular parameters could provide a more comprehensive understanding of hematological disorders in tribal populations across western India.

## Conclusions

This retrospective analysis underscores the indispensable role of BME in the diagnostic evaluation of hematological disorders among the tribal population of northern Maharashtra. Anemia, particularly microcytic and megaloblastic types, emerged as the most common indication for marrow evaluation, reflecting the persistent burden of nutritional deficiencies in this region. BMA and BMB demonstrated superior diagnostic accuracy compared to peripheral smear, especially in microcytic anemia and MDS, where statistically significant differences were observed. MPNs and PCDs, though less frequent, were reliably identified through marrow examination, reaffirming its value in diagnosing clonal and proliferative disorders. Importantly, several treatable conditions were detected that would likely have been missed on peripheral evaluation alone. These findings emphasize the need to strengthen hematology diagnostic infrastructure in underserved tribal areas, where timely access to BME can facilitate early diagnosis, appropriate management, and reduction of preventable hematologic morbidity.
